# Antibacterial and clinical effectiveness of a mouthwash with a novel active system of amine + zinc lactate + fluoride: a randomized controlled trial

**DOI:** 10.1007/s00784-023-05487-0

**Published:** 2024-01-13

**Authors:** Lorenzo Montesani, Luigi Montesani, Luis Mateo, Carlo Daep, Norbert Huber, Golnaz Isapour, Yun-Po Zhang

**Affiliations:** 1 Clinica Odontoiatria Montesani, Rome, Italy; 2 LRM Statistical Consulting, West Orange, NJ USA; 3 Colgate-Palmolive Technology Center, Piscataway, NJ USA; 4 Colgate-Palmolive Europe Sàrl, Therwil, Switzerland

**Keywords:** Mouthrinse, Antibacterial, Gingivitis, Dental plaque, Amine, Zinc lactate

## Abstract

**Objectives:**

To support the daily oral hygiene of patients experiencing gum inflammation, a new mouthwash was developed containing an amine + zinc lactate + fluoride system. In vitro and clinical efficacy was assessed using traditional methods as well as using novel site-specific and subject-specific analyses of the clinical data.

**Materials and methods:**

This mouthwash was evaluated in a 12-h biofilm regrowth assay against a negative control mouthwash and in a 6-month plaque and gingivitis clinical study as compared to a negative control mouthwash. Analyses of healthy versus inflamed sites, visible plaque versus non-visible plaque sites, as well as subject-level evaluations bring new perspectives to the overall performance of this mouthwash and its significance from a patient outcome perspective.

**Results:**

Studies demonstrated that this new mouthwash provided long-term (12-h) antibacterial activity after single application in vitro and reduced clinically all plaque and gingivitis parameters after 3 months and 6 months of use when compared to the negative control mouthwash. Examination of site-level and subject-level data determined that this mouthwash significantly increased the number of healthy sites in the oral cavity and significantly improved the gum health of subjects in the study, as compared to the negative control mouthwash.

**Conclusions:**

In vitro and clinical research has demonstrated the antibacterial and clinical benefits of this mouthwash containing an amine compound + zinc lactate + fluoride system.

**Clinical relevance:**

Our subject-specific and site-specific analyses provide the dental practitioner with tools that can be used to guide patients who suffer from gingivitis toward optimal product selection and use.

**Clinical trial registration:**

The trial was registered at ClinicalTrials.gov (reference no. NCT05821712).

## Introduction

Gingivitis is a site-specific inflammation of the gums that begins upon the accumulation of plaque biofilm in the oral cavity [1, 2]. This inflammation can be visually observed through erythema and swelling as well as the tendency for the site to bleed either spontaneously or upon mechanical probing [3]. Gingivitis is reversible; however, if left untreated, gingivitis can develop into periodontitis [4]. Mechanical removal of plaque via tooth brushing is one of the main methods to remove plaque, but it is not always successful to the extent that is needed [5].

Recent reviews have examined the incremental benefit of chemical plaque control in the management of pre-existing gingivitis [6, 7]. When included as part of an oral hygiene regimen, the use of a mouthwash containing antibacterial ingredients can be an effective intervention to improve plaque control and reduce gingival inflammation in the oral cavity [8]. These reviews concluded that the use of antibacterial mouthwashes as an adjunctive therapy to mechanical brushing provided significant reductions in gingivitis and plaque indices, supporting further clinical recommendations toward clinicians to consider recommending them in the context of daily oral hygiene for patients with gingivitis or periodontitis [6, 8]. It is further suggested that the use of these mouthwashes may be required, especially for those patients that are unable to effectively remove plaque by mechanical action alone [6].

To help clinicians decide whether a mouthwash provides the necessary efficacy in managing plaque and gingivitis, a 6-month randomized clinical trial should be conducted to evaluate efficacy and safety along with the assessment of the microbiological properties of the formulation [6].

An amine and stannous fluoride (ASF) system when delivered in mouthwashes was shown to reduce plaque in a 4-day plaque regrowth model [9] and when combined with zinc lactate (ZnL), was shown to reduce plaque in a 3-day regrowth model [10]. In a 4-week clinical study, the combination of amine and stannous fluoride in a mouthwash was shown to be more effective than a chlorhexidine mouthwash as measured by plaque index, gingival index, and gingival severity (bleeding) index in patients with chronic gingivitis [11]. The results from longer-term clinical studies on ASF mouthwashes have highlighted the potential of this combination as an adjunctive to brushing in patients with gum inflammation. Hoffmann et al. reported a reduction in plaque index, but not gingival index for ASF as compared to a water control rinse after 3 and 6 months of once daily use [12]. Schiffner et al. reported a gingivitis reduction for ASF as compared to control, but not in plaque after 6 months of once daily product use [13]. Finally, Zimmermann et al. reported statistically significant reductions in both plaque index and gingivitis index in those subjects who used an ASF mouthwash once daily for 7 months as compared to those subjects who used a placebo mouthwash [14].

ZnL has previously been combined with cetylpyridinium chloride (CPC) in a mouthwash. A regimen of tooth brushing with a fluoride toothpaste followed by use of this CPC + ZnL mouthwash was shown to reduce plaque and gingivitis after 4 weeks and 6 weeks significantly more than a regimen consisting of tooth brushing with a fluoride toothpaste and rinsing with an alcohol-free essential oil mouthwash or than tooth brushing with a fluoride toothpaste [15]. Thus, combining ZnL with an amine compound was used as a product development route, under the hypothesis that this association should provide further microbiological and clinical benefits.

Extensive clinical data show that patients suffering from plaque-induced gingivitis may benefit from multi-step oral care regimens to help control bacteria and produce measurable results, especially as tooth brushing alone often does not seem to produce the desired results. While mouthwashes are common adjuncts to tooth brushing, many products are perceived as not providing a good usage experience, altering taste, and causing tooth staining concerns [16–19], which can lead to less patient compliance.

To meet the needs of the patients who would benefit from effective plaque control while securing an optimal adherence profile, a new mouthwash has been developed containing an amine compound + ZnL + fluoride system. The evaluation of the clinical efficacy of this new mouthwash was preceded by evaluating its in vitro action with experiments on oral biofilm formation aiming to provide valuable insights into its effectiveness.

Previously, this mouthwash was evaluated in a series of laboratory tests including a short interval kill test (SIKT), a plaque glycolysis assay, and an aerobic biofilm model. These experiments determined that this new mouthwash killed planktonic bacteria over very short exposure times, and that the mouthwash affected both the viability and metabolic activity in model oral biofilms [20]. Additional experiments demonstrated the potential of key ingredients, such as the amine and ZnL, to inhibit oral biofilm formation [Schaeffer et al., 2023. In vitro effectiveness of a mouthwash with a novel amine compound + ZnL + fluoride active system. Manuscript in preparation].

In the current manuscript, the results from a 12-h (longer-term) biofilm regrowth assay are discussed. The results of this longer-term assay together with the previously published and unpublished in vitro results provided assurance that the mouthwash would perform as expected in terms of antibacterial efficacy and also provided the rationale to conduct a 6-month clinical study to examine the plaque and gingival effects of this new mouthwash in comparison to a negative control mouthwash. Furthermore, an analysis of healthy versus inflamed sites, an evaluation of sites with visible plaque as well as a subject-level analysis focused on the number of individuals improving plaque and the health of their gums, will bring a new perspective to the overall performance of this new mouthwash and its significance from a patient perspective.

## Materials and methods

### In vitro experiment

#### Test products

- Negative control: mouthwash containing 250 ppm fluoride in an alcohol-free base with identical color as test mouthwash (non-active ingredients: glycerin, propylene glycol, sorbitol, poloxamer, aroma, lactic acid, potassium sorbate, sucralose, CI 42051)
- AZF (test): mouthwash containing 0.2% zinc lactate, amine base compound and 250 ppm fluoride in an alcohol-free base (non-active ingredients: aqua, glycerin, xylitol, PVP, polyglyceryl-4 caprate, aroma, saccharin, sucralose, CI 42051)

Both mouthwashes were packed in identical white bottles and supplied by Colgate-Palmolive Europe Sàrl, Therwil, Switzerland.

#### 12-h biofilm regrowth

The 12-h biofilm regrowth experiments were conducted in the laboratories of the Colgate-Palmolive Company in Piscataway, NJ, USA. Saliva-derived biofilms were cultured vertically on HAP disks at 37 °C under 5% CO_2_. The biofilms were cultured in McBain media supplemented with 5 µg/mL (final concentration) hemin and 1 µg/mL final concentration vitamin K for a total of ~60 h. The media were replaced twice daily at ~12-h intervals. The resulting biofilm culture was treated with either the negative control mouthwash or the AZF mouthwash for 30 s under circular agitation (80 rpm). The biofilms were washed twice with sterile deionized water (dH_2_O) by dipping the treated biofilms 5 times in sterile deionized water each time. Following treatment, the biofilms were allowed to recover for approximately 2 h in sterile dH_2_O at 37 °C prior to harvesting the biofilms. Biofilms were dislodged from the HAP disks and resuspended in sterile dH_2_O by gently vortexing for 30 s. The treated bacterial suspensions were inoculated in 96-well polystyrene plates containing BHI broth supplemented with 2% yeast extract (final concentration) to a final optical density of 0.2 (absorbance = 610 nm) and final culture volume of 200 µL. The bacterial suspensions were cultured overnight at 37 °C. The bacterial density was measured (absorbance = 610 nm) hourly over 12 h. A total of 3 experiments were compiled and analyzed. Each experiment consisted of 4 biofilms per treatment group.

### Clinical study

#### Ethics approval

IRB approval was received from the Comitato Etico Romano, Rome, Italy. The clinical study was performed in accordance with the requirements specified in the 1964 Helsinki Declaration and its later amendments, ICH-GCP, and relevant local laws and regulations. All subjects signed an informed consent before any trial-related activities could begin. There were no amendments to the approved protocol.

#### Trial design and study location

This was a single center, randomized, parallel group, and triple-blind clinical trial that was conducted in healthy adult volunteers with mild gingivitis and lasted for 6 months. The examiner, subjects, and statistician were blinded to study product allocation. All subjects signed an informed consent before any study-related procedures were initiated. The trial was conducted between March 2021 and September 2021 at the Clinica Odontoiatrica Montesani, Rome, Italy.

#### Eligibility criteria

Subjects included in this clinical trial were between the ages of 18 and 70 (inclusive) and had to be in general good health. Subjects qualified for the trial with a mean gingival index score of at least 1.0 determined by the Löe-Silness gingival index and a mean plaque index score of at least 1.5 determined by the Turesky modification of the Quigley-Hein plaque index were enrolled in the study.

Potential subjects with the presence of orthodontic bands, tumors of the soft or hard tissues of the oral cavity, advanced periodontal disease (purulent exudate, tooth mobility, and/or extensive loss of periodontal attachment or alveolar bone), or five (5) or more carious lesions requiring immediate restorative treatment were excluded. In addition, subjects were not allowed to use any antibiotics within the 1 month prior to entry into the study. They were not allowed to participate in any other clinical study or test panel within the 1 month prior to entry into the study. They could not receive a dental prophylaxis in the 2 weeks prior to the baseline examination. Finally, subjects were excluded if they had a history of allergies to oral care/personal care consumer products or their ingredients, if their use of any prescription medicines might interfere with the study outcome, if there was an existing medical condition which prohibited eating or drinking for periods up to 4 h, if there was a history of alcohol or drug abuse, or if they were pregnant or lactating.

#### Sample size calculation

The sample size of 80 (40 per group) was determined based on the standard deviation for the response measures of 0.58, a significance level of *α* = 0.05, a 10% attrition rate, and an 80% level of power. The study was powered to detect a minimal statistically significant difference between study groups of 15%. The calculation utilized historical clinical data.

#### Randomization

A computer-generated list of random numbers was provided to the test site for use in assigning the subjects to one of the two mouthwashes.

#### Blinding

To mask their identity, the two study products were over-wrapped and coded by the sponsor. The person at the clinical site who oversaw the distribution of the study products and checking compliance was not involved in the clinical examinations.

#### Study interventions

Subjects were provided with either the AZF mouthwash or the negative control mouthwash and were instructed to rinse for 30 s with 15 mL of their assigned mouthwash twice daily, morning and evening, after brushing their teeth using an ordinary fluoride toothpaste (1450 ppm F) and a soft bristle toothbrush that were supplied by the study sponsor.

#### Gingivitis measurements

The primary measured responses were all gingival indices including whole-mouth gingivitis, gingival severity (bleeding), and gingival interproximal.

A Loe-Silness gingivitis index (GI) score from 0 to 3 was assigned by the examining dentist to all tooth surfaces using a dental light and dental mirror. A whole mouth mean score for each subject was determined by adding the values given by the examining dentist to each scorable surface and dividing that number by the total number of surfaces scored [3, 21].

- 0 = absence of inflammation
- 1 = mild inflammation—slight change in color and little change in texture
- 2 = moderate inflammation—moderate glazing, redness, edema, and hypertrophy
- 3 = severe inflammation—marked redness and hypertrophy. Tendency for spontaneous bleeding

Each tooth was scored on six surfaces: (1) mesio-facial; (2) mid-facial; (3) disto-facial; (4) mesio-lingual; (5) mid-lingual; and (6) disto-lingual. The maximum score per tooth, therefore, was 18.

Additionally, a gingival severity index was calculated that measured the proportion of scored tooth surfaces in the mouth whose assigned Loe-Silness gingivitis index scores were 2 or 3. Finally, a gingival interproximal index was calculated by summing the scores of the mesio-facial, disto-facial, mesio-lingual, and disto-lingual sites and dividing by the total number of evaluable sites.

#### Plaque measurements

Secondary measured responses included whole-mouth plaque, plaque severity, and plaque interproximal.

First using a red dye solution to disclose the plaque, a Quigley-Hein plaque index (PI) score from 0 to 5 was assigned to all scorable disclosed tooth surfaces using a dental light and dental mirror. A whole mouth score for each subject was determined by adding the values given by the dental examiner to each scorable surface and dividing that number by the total number of surfaces scored [22, 23].

- 0 = no plaque
- 1 = separate flecks of plaque at the cervical margin
- 2 = a thin, continuous band of plaque (up to 1 mm) at the cervical margin
- 3 = a band of plaque wider than 1 mm, but covering less than 1/3 of the side of the crown of the tooth
- 4 = plaque covering at least 1/3, but less than 2/3 of the side of the crown of the tooth
- 5 = plaque covering 2/3 or more of the side of the crown of the tooth

Each tooth was scored for supragingival plaque on six surfaces: (1) mesio-facial; (2) mid-facial; (3) disto-facial; (4) mesio-lingual; (5) mid-lingual; and (6) disto-lingual. The maximum score per tooth, therefore, is 30.

Additionally, a plaque severity index was calculated that measures the proportion of scored tooth surfaces in the mouth whose assigned Quigley-Hein plaque index scores were 3 or more. Finally, a plaque interproximal index was calculated by summing the scores of the mesio-facial, disto-facial, mesio-lingual, and disto-lingual sites and dividing by the total number of evaluable sites.

#### Site-level and subject-level analyses

Site-level and subject-level analyses were conducted on the whole mouth GI data and on the whole mouth PI data [2, 24].

##### Site-level analysis gingivitis

A site was considered healthy if it had a GI score of 0 or 1. A site was considered unhealthy (inflamed) if it had a score of 2 or 3. Additionally, those sites that scored 2 or 3 at baseline were further analyzed to determine the amount of improvement at each site over time.

##### Subject-level analysis gingivitis

The percentage of subjects for each treatment who have ≥ 90% healthy sites for gingival index was determined at 3 months and 6 months according to the definitions of the European Federation of Periodontology and the American Academy of Periodontology (AAP) [1].

##### Site-level analysis plaque

A site was considered healthy, i.e., plaque free or have no visible plaque, if it had a PI score of 0 or 1. A site was considered unhealthy and with visible plaque if it had a score of 2–5. Additionally, those sites that scored 2–5 at baseline were further analyzed to determine the amount of improvement at each site over time.

##### Subject-level analysis plaque

The percentage of subjects for each treatment who had ≥ 50% healthy sites for plaque index was determined at 3 months and 6 months.

#### Adverse events

All spontaneously reported adverse events (AEs) or abnormalities in the examination of the hard and soft oral tissues were recorded from the screening visit until the last visit.

### Statistical analyses

Results of the 12-h biofilm regrowth were analyzed using a 1-way analysis of variance (ANOVA) using Tukey’s multiple comparison test.

Statistical analyses were performed separately for the gingivitis assessments and the dental plaque assessments. Comparison of the treatment groups with respect to gender were performed using a chi-square analysis and for age using an independent *t*-test. Comparisons of the treatment groups with respect to baseline GI and PI scores were performed using ANOVA. Within-treatment comparisons of the baseline versus follow-up GI and PI scores were performed using paired *t*-tests. Comparisons of the treatment groups with respect to baseline-adjusted GI and PI scores at the follow-up examinations were performed using analyses of covariance (ANCOVA). All statistical tests of hypotheses were two sided and employed a level of significance of *α* = 0.05.

## Results

### 12-h biofilm regrowth kinetics

**Fig. 1:**
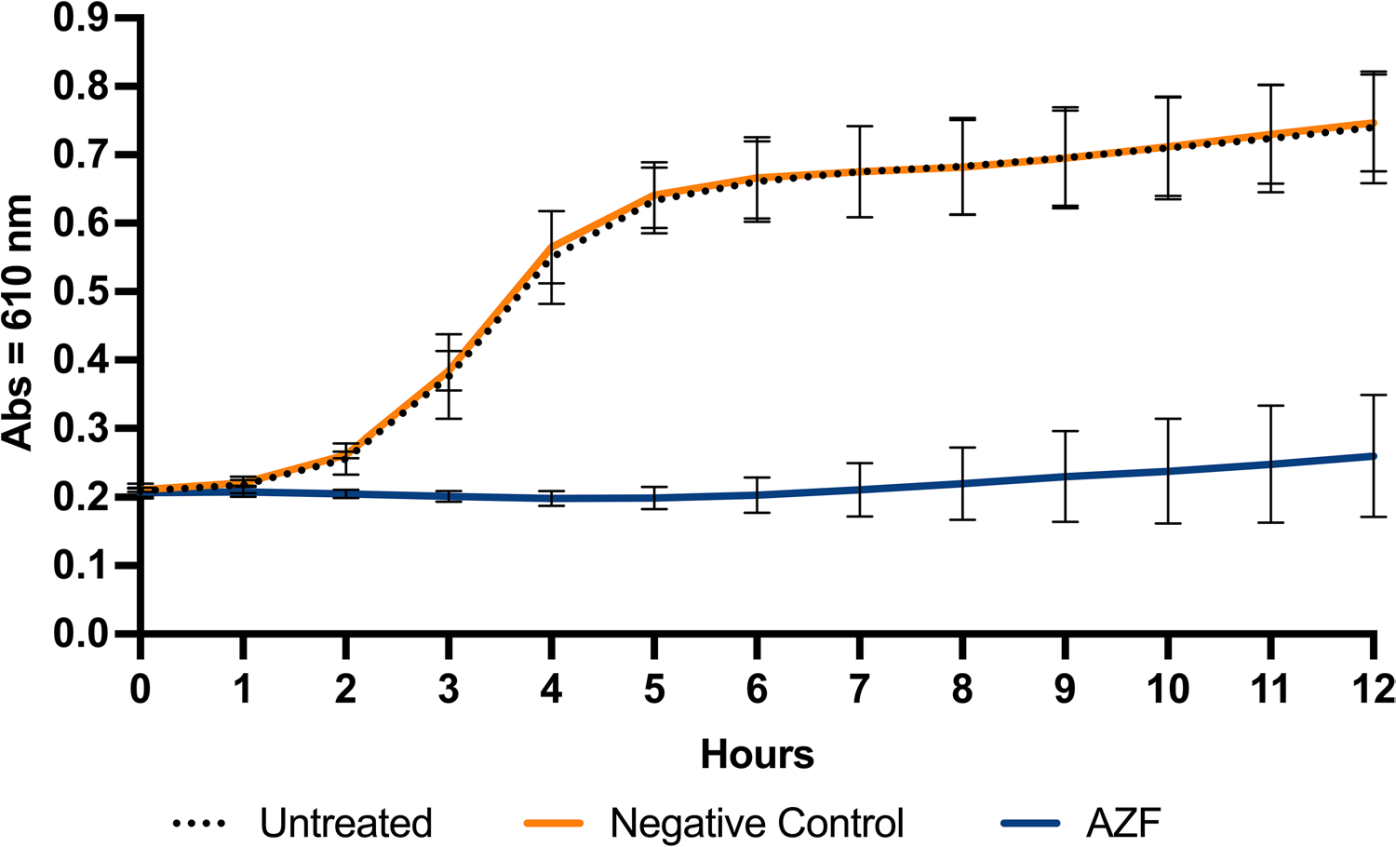
Comparison of 12-h bacteria regrowth post-treatment of biofilm with AZF mouthwash to biofilm treated with negative control mouthwash and to the untreated biofilm. Each data point is an average over 3 experiments with 4 data points obtained per experiment. Bacterial growth was measured using optical density (OD) via absorbance at 610 nm. Bacterial growth on biofilm treated with the AZF mouthwash was statistically significantly (*p* < 0.0001) lower than either the bacterial growth on the untreated biofilm or on the negative control treated biofilm starting at 2 h and continuing for all later timepoints

The regrowth study showed that relative to the untreated biofilms and biofilms treated with the negative control mouthwash, the regrowth of bacteria derived from the biofilms treated with the AZF mouthwash was significantly reduced or inhibited. This is based on the reduced bacterial culture absorbance over the 12-h period as measured each hour (Fig. 1). Relative to the untreated biofilms and the biofilms treated with the negative control mouthwash, biofilms that were treated with the AZF mouthwash significantly inhibited bacterial regrowth (*p* < 0.001) beginning at 2 h and continuing for 12 h. No observed difference in antibacterial activity was documented for biofilms treated with the negative control mouthwash when compared with the untreated biofilm at all timepoints.

### Clinical study

#### Subject flow

**Fig. 2:**
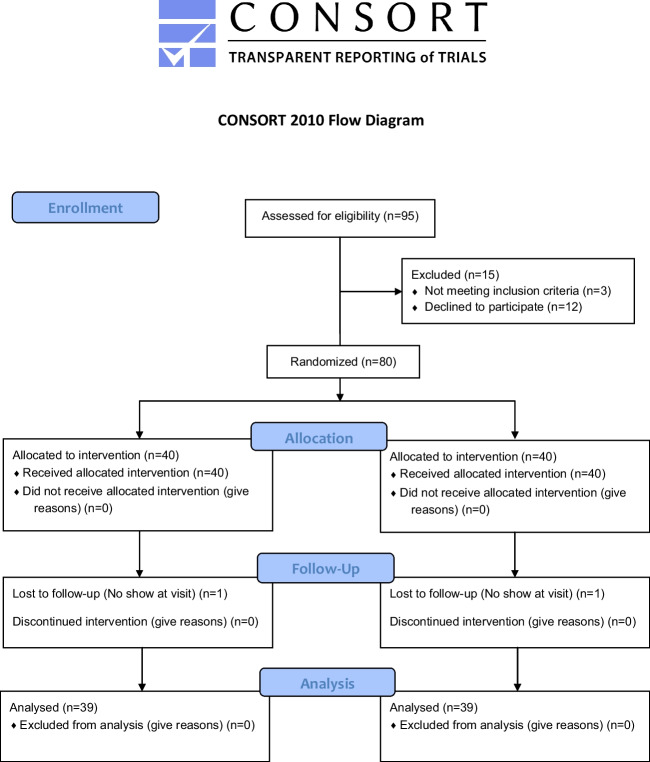
CONSORT diagram

A total of 95 individuals were assessed for study eligibility. Fifteen subjects were excluded from the study: 3 did not meet the inclusion criteria, and 12 declined to participate. Eighty individuals were randomized into the clinical study (Fig. 2). Forty subjects were assigned to each of the treatment groups. One subject from each group was dropped from the study because of failure to attend a study visit and was not product related. The data analyses were performed on this per-protocol population. Fifty-one percent of the subjects were male with the age range of the subjects ranging from 21 to 67 with an average age of 46.3 years.

#### Clinical efficacy gingivitis

Table 1 presents the baseline-adjusted means after 3 months and 6 months of product treatment. There were no statistically significant differences between the two treatment groups for any of the gingival indices at baseline (data not shown). At the 3-month and 6-month examinations, both treatments provided a statistically significant reduction (*p* < 0.001) as compared to baseline. The percentage differences between the two groups after 3 months and 6 months of treatment are shown in Table 1 for all three gingival indices. These between product differences are statistically significant (*p* < 0.001) at both 3 months and 6 months. After 6 months of treatment, these percentage differences resulted in the AZF mouthwash being 6.2 × more effective for gingivitis, 5.3 × more effective for gingival severity (or bleeding), and 6.1 × more effective for gingival interproximal as compared to the negative control mouthwash. All these differences for gingivitis are statistically significant (*p* < 0.001).

**Table 1:** Statistical parameters for comparisons made between treatment groups at 3-month and 6-month intervals, using baseline-adjusted means

	Mean (S.E.)		% difference	*p* value
	AZF mouthwash	Negative control mouthwash		
Gingival index				
3 months	1.27 (0.01)	1.54 (0.01)	17.5%	*p* < 0.001
6 months	1.04 (0.01)	1.50 (0.01)	30.7%	*p* < 0.001
Gingival severity				
3 months	0.23 (0.01)	0.40 (0.01)	42.5%	*p* < 0.001
6 months	0.07 (0.01)	0.37 (0.01)	81.1%	*p* < 0.001
Gingival interproximal				
3 months	1.26 (0.02)	1.53 (0.02)	17.6%	*p* < 0.001
6 months	1.04 (0.01)	1.49 (0.01)	30.2%	*p* < 0.001
Plaque index				
3 months	1.74 (0.02)	2.07 (0.02)	15.9%	*p* < 0.001
6 months	1.46 (0.02)	2.02 (0.02)	27.7%	*p* < 0.001
Plaque severity				
3 months	0.22 (0.01)	0.32 (0.01)	31.3%	*p* < 0.001
6 months	0.13 (0.01)	0.31 (0.01)	58.1%	*p* < 0.001
Plaque interproximal				
3 months	1.75 (0.02)	2.07 (0.02)	15.5%	*p* < 0.001
6 months	1.65 (0.02)	2.03 (0.02)	28.1%	*p* < 0.001

#### Clinical efficacy plaque

Table 1 presents the baseline-adjusted means after 3 months and 6 months of product treatment. There were no statistically significant differences between the two treatment groups for any of the plaque indices at baseline (data not shown). At the 3-month and 6-month examinations, both treatments provided a statistically significant reduction (*p* < 0.001) compared to baseline. The percentage differences between the two groups after 3 months and 6 months of treatment are shown in Table 1 for all three plaque indices. These differences are statistically significant (*p* < 0.001) at both 3 months and 6 months. After 6 months of treatment, these percentage differences resulted in the AZF mouthwash being 5.9 × more effective for plaque, 6.8 × more effective for plaque severity, and 6.1 × more effective for plaque interproximal compared to the negative control mouthwash. All these differences for plaque are statistically significant (*p* < 0.001).

#### Healthy sites analysis

##### Gingivitis

**Fig. 3:**
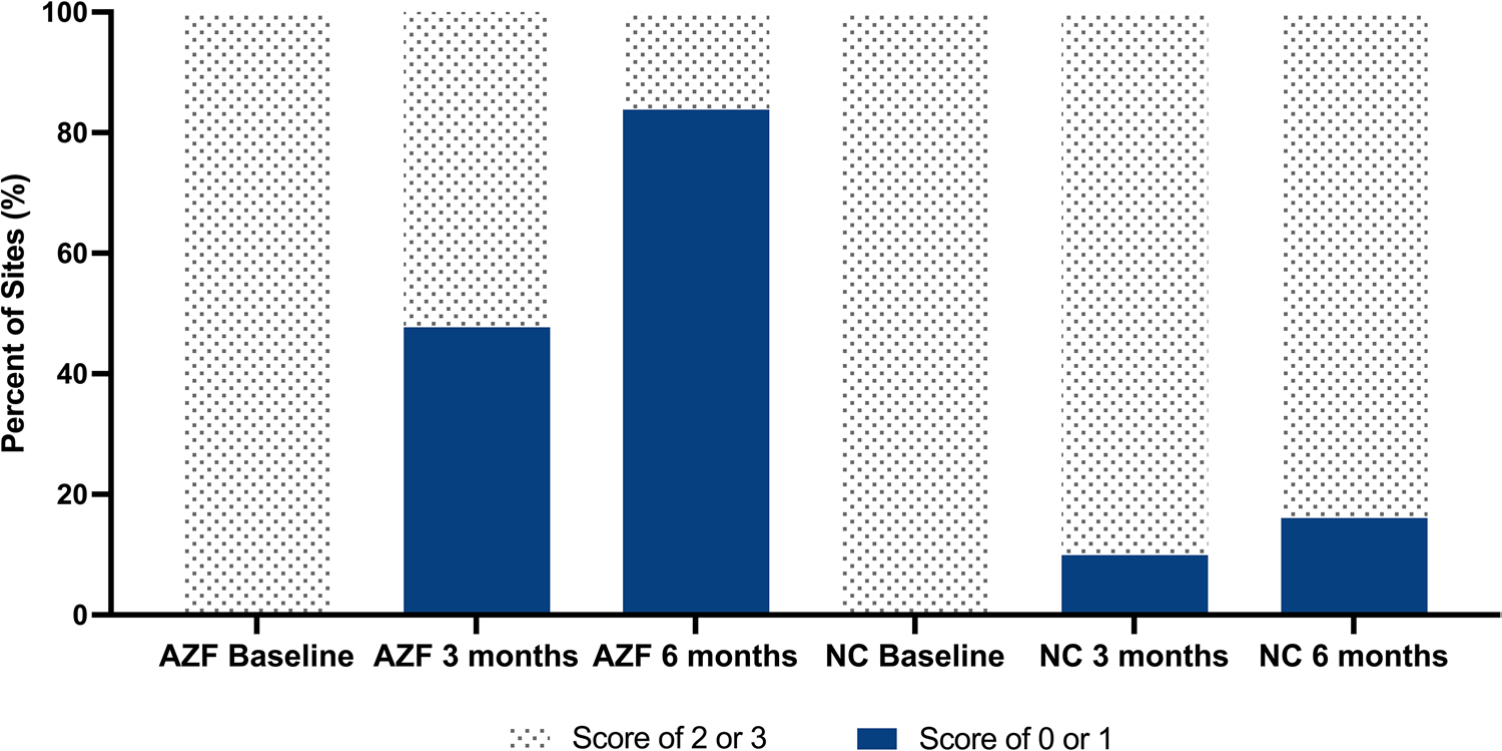
Site-level analysis of percentage of sites that were healthy for gingivitis. A site was considered healthy if it had a GI score of 0 or 1. A site was considered inflamed for gingivitis if it had a GI score of 2 or 3

For those who used the AZF mouthwash, the number of subjects (% of group) who had at least 90% healthy sites increased from 4 (10.3%) at 3 months to 28 (71.8%) at 6 months for gingival index. On the other hand, 0 subjects (0%) who used the negative control mouthwash had at least 90% healthy sites at either 3 months or 6 months. The site level analysis is shown in Fig. 3. At baseline, the total number of inflamed sites as scored for gingivitis was 2902 for those who used the AZF mouthwash and 2872 for those who used the negative control mouthwash. After 6 months of using the AZF mouthwash, more than 80% of the inflamed sites had become healthy as compared to only 16% of the sites for those who used the negative control mouthwash. Overall, 7 out of 10 subjects achieved healthy gingival sites after 6 months of use of the AZF mouthwash with a ≥ 50% improvement in gum health in 9 out of 10 subjects. On the other hand, none of the subjects using the negative control mouthwash achieved healthy gingival sites.

##### Plaque

**Fig. 4:**
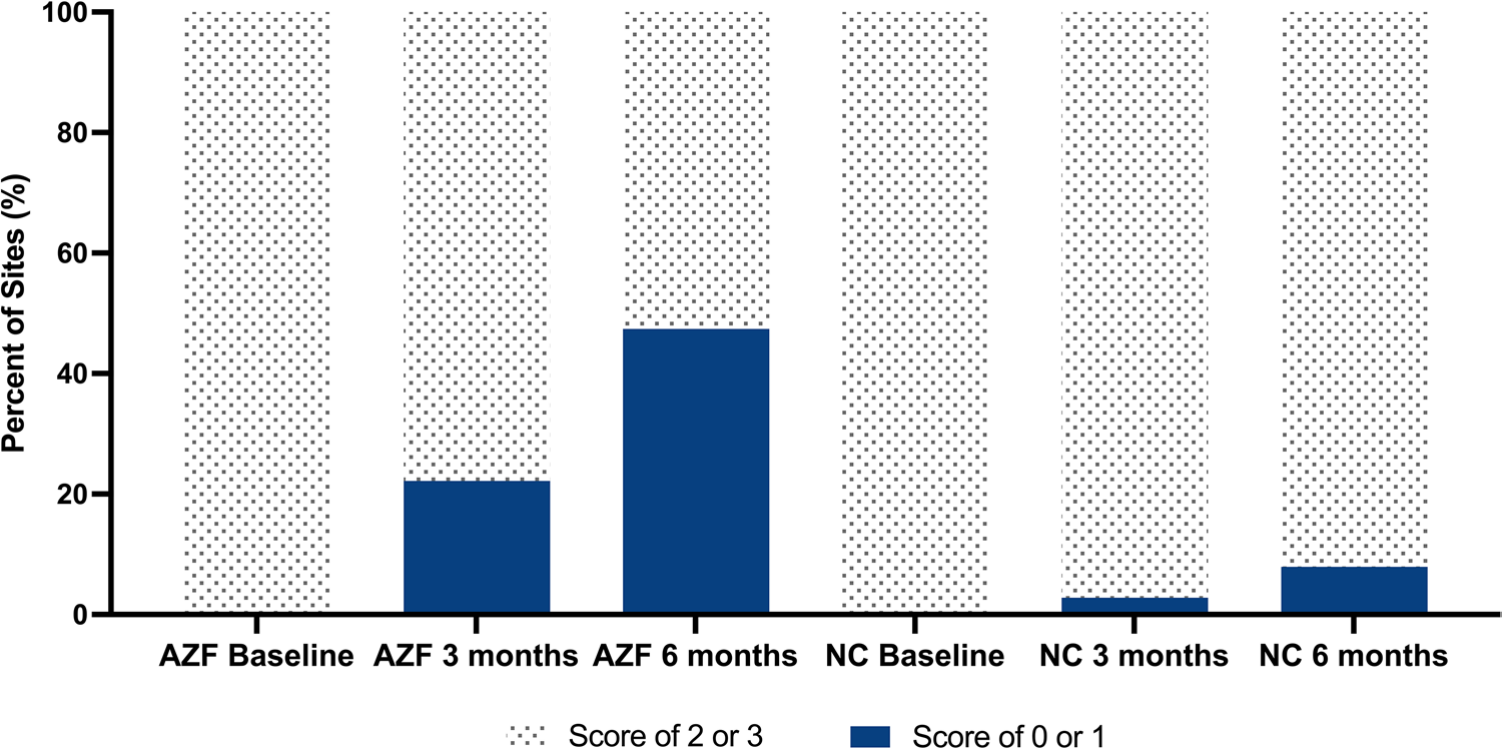
Site-level analysis of percentage of sites that were healthy for plaque. A site was considered healthy for plaque if it had a PI score of 0 or 1. A site was considered to have visible plaque if it had a score of 2–5

For those who used the AZF mouthwash, the number of subjects (% of group) at 6 months who had at least 50% of sites that contained no visible plaque or plaque free was 13 subjects (33.3%) at 6 months as compared to 0 subjects (0%) who used the negative control mouthwash. The site level analysis is shown in Fig. 4. At baseline, the total number of sites with visible plaque as scored for plaque was 2180 for those who used the AZF mouthwash and 2250 for those who used the negative control mouthwash. After 6 months of using the AZF mouthwash, more than 47% of the sites with visible plaque had become healthy as compared to less than 8% of the sites for those who use the negative control mouthwash.

## Safety results

During the clinical trial, there were no adverse effects noted by the examiner on the oral hard or soft tissues. Neither were any adverse effects reported by the subjects when questioned.

## Discussion

Plaque-induced gingivitis is a very common disease, with recent estimates suggesting that it affects more than 75% of the population [25–28]. Mechanical plaque control in conjunction with an antibacterial toothpaste is often used to help control plaque and gingivitis [29, 30]. Regular use of antibacterial mouthwashes acts as a supplement to reduce the plaque levels beyond that seen by tooth brushing alone. Clinical efficacy of these mouthwashes against plaque-induced gingivitis, as well as their safety, should be evaluated in long-term (i.e., 6 months) randomized controlled trials (RCT). Thus, the present study aimed to evaluate this new mouthwash in a 6-month RCT.

Three reviews have examined the effectiveness of the most common mouthwashes active ingredients [31–33]. These reviews concluded that for the mouthwashes containing the most common antibacterial ingredients, i.e., chlorhexidine gluconate, cetylpyridinium chloride, and essential oils, there is the necessary scientific support regarding their beneficial activity against plaque and gingivitis. However, there was no meaningful discussion of mouthwashes based on amine actives due to the lack of published literature or its lack of presence in the marketplace. In addition, Takenaka et al. concluded that a meta-analysis of amine fluoride mouthwash studies produced inconsistent results that indicated no difference from placebo rinses. Their analysis called into question their efficacy against oral biofilm control [34].

Thus, the present study aimed to evaluate this new mouthwash to increase the evidence for amine-based mouthwashes in both a biofilm regrowth model and a 6-month RCT. Prior to the RCT, the formula was subjected to a number of laboratory experiments to establish the short-term and long-term antibacterial effect of the mouthwash.

To supplement the previously published in vitro results [20], the longevity of antibacterial effectiveness of the mouthwashes was evaluated in a 12-h biofilm regrowth model (Fig. 1). The AZF mouthwash was effective at inhibiting bacterial regrowth beginning as soon as 2 h after the single application and extending for up to 12 h. Taken together, the short-term and long-term in vitro data demonstrated that the combination of an amine compound + ZnL in a mouthwash formulation provided considerable antibacterial efficacy. Based on these results, a 6-month RCT was undertaken to assess the clinical efficacy in terms of gingivitis and plaque accumulation of the AZF mouthwash.

The results of this RCT showed that all plaque and gingivitis parameters (i.e., whole mouth, severity, and interproximal) improved relatively to baseline for both the AZF mouthwash and the negative control mouthwash as measured after 3 months and 6 months of product use. However, the AZF mouthwash showed a significantly superior improvement in all plaque and gingivitis parameters as compared to the negative control mouthwash after 3 months and 6 months. In addition, there were no safety concerns reported or noted during the study.

These results are based on the average plaque and gingivitis indices and speak to the benefit that would be observed by a population as a whole and provide no information on the effect on individual subjects and tooth sites. Thus, novel subject-level and site-level analyses have been conducted on the data. These types of analyses were retrospectively applied in a meta-analysis [24], but this is the first time to our knowledge that these analyses have been applied directly to clinical data and reported as such.

The novel subject-level and site-level analyses of the whole mouth GI and the whole mouth PI data indicate an overall improvement in oral health because of using the AZF mouthwash. These analyses allow the dental practitioner to understand the product’s efficacy for an individual patient. In the current study, the number of subjects who had at least 90% healthy gingival sites increased from 4 out of 39 at 3 months to 28 out of 39 at 6 months. This means that almost 75% of the subjects have shown a benefit from using the AZF mouthwash, while no subjects using the negative control mouthwash presented with at least 90% healthy sites. Similarly, on a site level, 4 out of every 5 scorable sites improved from inflamed to healthy for users of the AZF mouthwash as compared to only 1 out of 6 of the sites for users of the negative control mouthwash.

A similar result was seen with the analyses of the plaque data. At 6 months, 13 subjects (33.3%) who used the AZF mouthwash had at least 50% of the sites free of visible plaque or plaque free, while no subjects who used the negative control mouthwash reached this cut-off value. On a site level, at 6 months 47% of the sites with previously visible plaque had become healthy (plaque free or with no visible plaque) for users of the AZF mouthwash as compared to less than 8% of the sites for users of the negative control mouthwash. Once again, the dental practitioner can use these results for the benefit of the patient.

Previous studies on amine-based mouthwashes have produced mixed results. In one publication, plaque was reduced but not gingivitis [12], in a second, gingivitis was reduced but not plaque [13], while in a third both plaque and gingivitis were reduced [11, 14]. Variations in study product along with differing study populations and design could be reasons for the observed difference in the reported results as compared to each other and as compared to our results. Other factors to consider include the age range of the subjects, the number of required teeth, and baseline gingival conditions.

Overall, the data demonstrates that a mouthwash containing an amine compound + ZnL + fluoride system has long-term (12-h) in vitro antibacterial activity. Additionally, the efficacy of this mouthwash against plaque and plaque-induced gingivitis has been demonstrated in a 6-month RCT, in comparison with mouthwash with no antibacterial efficacy. Additional analyses of healthy sites and the number of subjects benefiting from the effect of the mouthwash in reducing gingivitis provide additional information to the dental practitioner and confirmed the overall benefit of this new formulation.
