# Correction to: Antibacterial and clinical efectiveness of a mouthwash with a novel active system of amine + zinc lactate + fuoride: a randomized controlled trial

**DOI:** 10.1007/s00784-024-05575-9

**Published:** 2024-02-29

**Authors:** Lorenzo Montesani, Luigi Montesani, Luis Mateo, Carlo Daep, Norbert Huber, Golnaz Isapour, Yun-Po Zhang

**Affiliations:** 1 Clinica Odontoiatria Montesani, Rome, Italy; 2 LRM Statistical Consulting, West Orange, NJ USA; 3 Colgate-Palmolive Technology Center, Piscataway, NJ USA; 4 Colgate-Palmolive Europe Sàrl, Therwil, Switzerland

**Correction to: Clinical Oral Investigations.** 10.1007/s00784-023-05487-0

The article “Antibacterial and clinical efectiveness of a mouthwash with a novel active system of amine+zinc lactate+fuoride: a randomized controlled trial”, written by Lorenzo Montesani, Luigi Montesani, Luis Mateo, Carlo Daep, Norbert Huber, Golnaz Isapour and Yun Po Zhang, was originally published Online First without Open Access. After publication in volume 28, issue 1, page 963–972 the author decided to opt for Open Choice and to make the article an Open Access publication. Therefore, the copyright of the article has been changed to © The Author(s) 2024 and the article is forthwith distributed under the terms of the Creative Commons Attribution 4.0 International License, which permits use, sharing, adaptation, distribution and reproduction in any medium or format, as long as you give appropriate credit to the original author(s) and the source, provide a link to the Creative Commons licence, and indicate if changes were made. The images or other third party material in this article are included in the article’s Creative Commons licence, unless indicated otherwise in a credit line to the material. If material is not included in the article’s Creative Commons licence and your intended use is not permitted by statutory regulation or exceeds the permitted use, you will need to obtain permission directly from the copyright holder. To view a copy of this licence, visit http://creativecommons.org/licenses/by/4.0.

The original article has been corrected.

